# Inflammatory factor TNF-α promotes the growth of breast cancer via the positive feedback loop of TNFR1/NF-κB (and/or p38)/p-STAT3/HBXIP/TNFR1

**DOI:** 10.18632/oncotarget.16873

**Published:** 2017-04-06

**Authors:** Xiaoli Cai, Can Cao, Jiong Li, Fuquan Chen, Shuqin Zhang, Bowen Liu, Weiying Zhang, Xiaodong Zhang, Lihong Ye

**Affiliations:** ^1^ State Key Laboratory of Medicinal Chemical Biology, Department of Biochemistry, College of Life Sciences, Nankai University, Tianjin 300071, China; ^2^ State Key Laboratory of Medicinal Chemical Biology, Department of Cancer Research, College of Life Sciences, Nankai University, Tianjin 300071, China

**Keywords:** TNF-α, HBXIP, STAT3, growth, breast cancer

## Abstract

In the connection between inflammation and cancer development, tumor necrosis factor-alpha (TNF-α) contributes to the tumorigenesis. However, the underlying mechanism remains poorly understood. In this study, we report that TNF-α enhances the growth of breast cancer through up-regulation of oncoprotein hepatitis B X-interacting protein (HBXIP). Our data showed that the levels of TNF-α were positively related to those of HBXIP in clinical breast cancer tissues. Moreover, TNF-α could up-regulate HBXIP in breast cancer cells. Interestingly, silencing of TNF-α receptor 1 (TNFR1) blocked the effect of TNF-α on HBXIP. Mechanistically, we revealed that TNF-α could increase the activities of HBXIP promoter through activating transcriptional factor signal transducer and activator of transcription 3 (STAT3). In addition, nuclear factor kappa B (NF-κB) and/or p38 signaling increased the levels of p-STAT3 in the cells. Strikingly, HBXIP could also up-regulate TNFR1, forming a positive feedback loop of TNFR1/NF-κB (and/or p38)/p-STAT3/HBXIP/TNFR1. Notably, TNF-α was able to up-regulate TNFR1 through driving the loop. In function, we demonstrated that the knockdown of HBXIP remarkably abolished the growth of breast cancer mediated by TNF-α *in vitro* and *in vivo*. Thus, we conclude that TNF-α promotes the growth of breast cancer through the positive feedback loop of TNFR1/NF-κB (and/or p38)/p-STAT3/HBXIP/TNFR1.Our finding provides new insights into the mechanism by which TNF-α drives oncoprotein HBXIP in the development of breast cancer.

## INTRODUCTION

The links between inflammation and cancer become a hot topic for cancer. However, the underlying molecular mechanism remains unclear. The cancer-related inflammation involving in the progression of cancer includes the presence of inflammatory cells and inflammatory mediators which including chemokines, cytokines and prostaglandins in cancer microenvironment [1, 2]. Among the numerous cytokines, tumor necrosis factor-alpha (TNF-α) is an important multifunctional inflammatory cytokine with pleiotropic actions, involving the regulation of apoptosis, survival and immune responses [3–5]. It has been reported that various kinds of cells can release TNF-α, such as fibroblasts, Kupffer cells, keratinocytes, the infiltrating inflammatory cells, including tumor-infiltrating macrophages, and tumor cells which including B-cell lymphoma and colon carcinomas cells [6, 7]. TNF-α is able to increase the production of cytokines, proteases and growth-factors through binding to its receptors including TNF-α receptor 1 (TNFR1) and TNF-α receptor 2 (TNFR2) [8]. TNFR2 is restrictively expressed in immune cells and mediates limited biological functions, whereas TNFR1 is ubiquitously expressed in human tissues and serves as a major signaling receptor for TNF-α [9]. However, whether TNF-α can up-regulate its receptors is unclear. Inflammatory factor TNF-α is able to promote the growth, invasion, metastasis and lymphangiogenesis in cancer development [6, 7, 10–13]. As a tumor promoter, TNF-α activates Noxo1 and Gna14 to enhance gastric tumorigenesis [11]. However, the role of TNF-α in the development of breast cancer is not well documented.

Nuclear factor kappa B (NF-κB) responded to the activation of TNFR-associated death domain protein (TRADD) mediating the recruitment of protein complex leads to cell survival and release of inflammatory mediators [14]. NF-κB acts as a key regulator coordinating the balance between innate immunity and inflammation [15]. It has been reported that, in most cell types, the activation of TNFR1 can trigger canonical or noncanonical NF-κB-dependent transcription regulation of genes encoding pro-survival and pro-inflammatory molecules [16]. The activation of NF-κB mediated by TNFR1 promotes the proliferation of squamous cells in head and neck [10]. NF-κB and p38/MAPK signaling synergistically promote the production of cytokines IL-6 induced by TNF-α [17]. Like other MAPK family members, p38 as a phosphokinase initiates the signaling cascades to promote cell growth, differentiation, apoptosis, and responds to inflammation or stress [18]. Signal transducer and activator of transcription 3 (STAT3) mediates the transduction of signals from cytokine receptors to the nucleus [19]. The constitutively activation of STAT3 is a point of convergence for numerous oncogenic signaling pathways in cancers [20]. In condition of TNF-α administration, TNFR1 results in the activation of canonical or non-canonical NF-κB pathway, followed by inducing the IL-6/JAK1/2/STAT3 signaling [21, 22]. However, the significance of TNF-α in regulation of TNFR1/NF-κB (and/or p38)/p-STAT3 in breast cancer is elusive.

Mammalian hepatitis B X-interacting protein (HBXIP, or termed as LAMTOR5) is originally identified for its binding to the C terminus of the hepatitis B virus X protein [23]. HBXIP serves as a novel ragulator component that is required for mTORC1 activation [24]. HBXIP can collaborate with survivin to control cell apoptosis and division, and also serves as a modulator of centrosome dynamics and cytokinesis to mediate cell growth [25, 26]. Our group has reported that HBXIP is highly expressed in breast cancer, displaying a function of oncoprotein [27–32]. Recently, we have showed that HBXIP can act as a coactivator of transcription factors, such as c-myc, SREBP-1c, to facilitate the proliferation and lipid metabolism in breast cancer [33, 34]. However, the effect of TNF-α on HBXIP in breast cancer remains poorly understood.

In this study, we are interested in the significance of inflammatory factor TNF-α on oncoprotein HBXIP in the links between inflammation and cancer. Strikingly, we found that TNF-α was able to up-regulate HBXIP through driving a positive feedback loop of NF-κB (and/or p38)/p-STAT3/HBXIP/TNFR1, resulting in the enhancement of growth of breast cancer. Our finding provides new insights into the mechanism by which TNF-α up-regulates HBXIP in the development of breast cancer.

## RESULTS

### TNF-α administration is able to promote the proliferation of breast cancer cells through activating TNFR1

**Figure 1:**
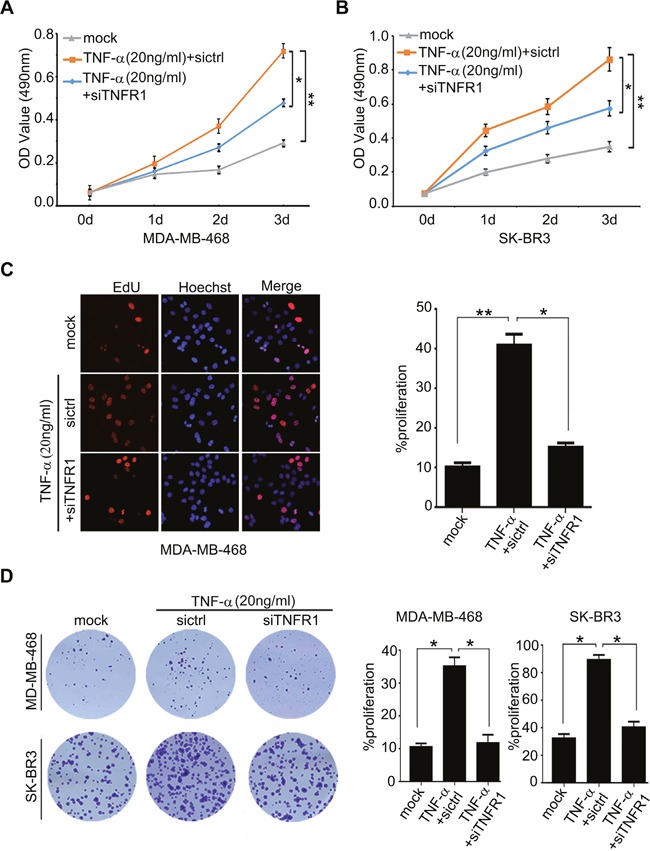
TNF-α administration is able to promote the proliferation of breast cancer cells through activating TNFR1 **(A, B)** Effect of TNF-α on cell proliferation was measured by MTT assays in MDA-MB-468 and SK-BR3 which were added into 20 ng/ml TNF-α and transiently transfected with si-TNFR1, respectively. **(C)** Effect of TNF-α on cell proliferation was determined by EdU assays in MDA-MB-468 cells added into 20 ng/ml TNF-α and transiently transfected with si-TNFR1. **(D)** Effect of TNF-α and si-TNFR1 on the clonogenicity of MDA-MB-468 and SK-BR3 cells was examined by monolayer colony formation assays. Error bars represent ±s.d., *p < 0.05, **p < 0.01, Student's *t* test. All experiments were repeated at least 3 times.

It has been reported that TNF-α promotes gastric tumorigenesis and tumor lymphangiogenesis [7, 11]. Therefore, we supposed that TNF-α might be involved in the proliferation of breast cancer cells. According to the report that the treatment with 10 ng/ml or 100 ng/ml TNF-α could induce the increase of cancer stem cell populations and tumor lymphangiogenesis [7, 35]. We showed that the treatment with 20 ng/ml TNF-α was able to enhance the proliferation in MDA-MB-468 and/or SK-BR3 cells by using MTT, EdU incorporation and colony formation assays. Furthermore, silencing of TNFR1 by siRNA significantly inhibited the proliferation of MDA-MB-468 or SKBR-3 cells induced by TNF-α (Figure 1A-1D), suggesting that TNF-α promotes cell proliferation of breast cancer through activating TNFR1. The expression of TNFR1 was validated by Western blot analysis in the cells (Supplementary Figure 1A). To rule out the possibility of apoptosis mediated by 20 ng/ml TNF-α, we examined cell apoptosis by using Annexin-V-PI assays. As expected, we observed that the treatment of 20ng/ml TNF-α failed to induce apoptosis in the cells, suggesting that our system can rule out the possibility of apoptosis induced by 20 ng/ml TNF-α (Supplementary Figure 1B). In addition, we observed that siTNFR1 could significantly induce apoptosis, suggesting that knockdown of TNFR1 can block the effect of TNF-α on promotion of cell proliferation in breast cancer. Thus, we conclude that inflammatory cytokine TNF-α promotes the proliferation of breast cancer cells through activating its receptor TNFR1.

### TNF-α is positively correlated with HBXIP in clinical breast cancer tissues and up-regulates HBXIP in breast cancer cells

**Figure 2:**
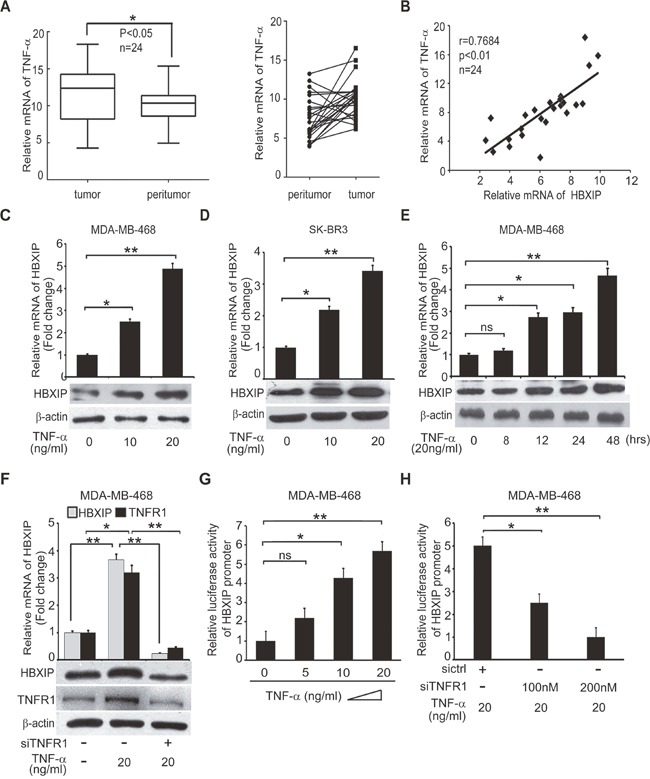
TNF-α is positively correlated with HBXIP in clinical breast cancer tissues and up-regulates HBXIP in breast cancer cells **(A)** The relative mRNA levels of TNF-α were detected by qRT-PCR in clinical breast cancer tissues (n=24) (*p<0.05, Wilcoxon's signed-rank test). **(B)** The relative mRNA levels of HBXIP and TNF-α were detected by qRT-PCR and normalized against GAPDH in above samples (Spearman's correlation = 0.7684, **p < 0.01). **(C, D)** The mRNA and protein levels of HBXIP were measured by qRT-PCR and Western blot analysis in MDA-MB-468 and SK-BR3 cells treated with 10 ng/ml, 20 ng/ml TNF-α, respectively. **(E)** The expression of HBXIP in mRNA and protein levels were measured by qRT-PCR and Western blot analysis in MDA-MB-468 cells treated with 20 ng/ml TNF-α in a time course (0-48 hours). **(F)** The expression levels of HBXIP and TNFR1 were measured by qRT-PCR and Western blot assays in MDA-MB-468 cells treated with 20 ng/ml TNF-α and transfected with siTNFR1. **(G, H)** Dual luciferase reporter gene assays were performed to detect the relative activities of HBXIP promoter in MDA-MB-468 cells added into 5 ng/ml, 10 ng/ml and 20 ng/ml TNF-α and/or transiently transfected with 100 nM, 200 nM siTNFR1. Error bars represent ±s.d., *p<0.05, **p< 0.01, Student's *t* test. All experiments were performed at least 3 times.

TNF-α is able to promote the development of cancer through activating cancer-related pathways or up-regulating Noxo1 and Gna14 [11]. Previously, we demonstrated that the oncoprotein HBXIP promoted the development of breast cancer [27–30, 33, 34]. Accordingly, we are interested in whether TNF-α promotes the development of breast cancer through up-regulating HBXIP. Quantitative real-time PCR (qRT-PCR) analysis revealed that the mRNA levels of TNF-α were higher in 24 cases of clinical breast tumor tissues relative to their adjacent peritumor tissues (*p<0.05, Wilcoxon's signed-rank test, Figure 2A and Supplementary Table 1). Moreover, we observed that the mRNA levels of TNF-α were positively correlated with those of HBXIP in above tissues (r = 0.7684, **p< 0.01, Pearson's correlation, Figure 2B), raising the possibility that TNF-α may up-regulate HBXIP in breast cancer. As expected, we validated that TNF-α was able to up-regulate the expression of HBXIP, at the levels of mRNA and protein in MDA-MB-468 and/or SK-BR3 cells in a dose or time-dependent manner (Figure 2C-2E and Supplementary Figure 2A-2C). However, TNF-α failed to work in MDA-MB-468 cells when TNFR1 was knockdown by siRNA (Figure 2F and Supplementary Figure 2D), suggesting that TNF-α up-regulates HBXIP depending on TNFR1 in breast cancer cells. Next, we showed that TNF-α could activate HBXIP promoter in MDA-MB-468 cells in a dose-dependent manner (Figure 2G). Conversely, silencing of TNFR1 significantly abolished the event (Figure 2H). Taken together, we conclude that TNF-α is positively related to HBXIP in clinical breast cancer tissues and up-regulates HBXIP in breast cancer cells.

### TNF-α activates HBXIP promoter through activating transcription factor STAT3

**Figure 3:**
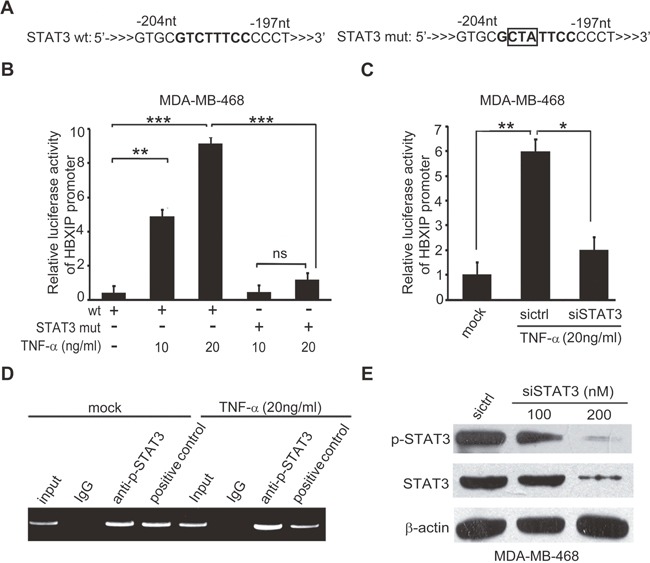
TNF-α activates HBXIP promoter through activating transcription factor STAT3 **(A)** The illustration of wild or mutant sequence responsive to STAT3 in HBXIP promoter was shown. **(B)** The relative activities of HBXIP promoter containing wild type (wt) or mutant type of STAT3 (STAT3 mut) binding site were detected by luciferase reporter gene assays in MDA-MB-468 cells administrated with 10 ng/ml, 20 ng/ml TNF-α. **(C)** The relative activities of the promoter of HBXIP were detected by luciferase reporter gene assays in MDA-MB-468 cells administrated 20 ng/ml TNF-α and transiently transfected with STAT3 siRNA. **(D)** The enrichment of transcription factor STAT3 in the HBXIP promoter was detected by CHIP assays by using antibody against p-STAT3 in MDA-MB-468 cells upon TNF-α treatment. **(E)** The levels of STAT3, p-STAT3 were detected by Western blot analysis in MDA-MB-468 cells transfected with STAT3 siRNAs. Error bars represent ±s.d., *p< 0.05, **p< 0.01, ***p< 0.001, Student's *t* test. All experiments were repeated at least 3 times.

To gain insight into the mechanism by which TNF-α activates the promoter of HBXIP, we predicted the transcription factor binding sites in HBXIP promoter by Gene Promoter Miner (http://gpminer.mbc.nctu.edu.tw/). Interestingly, we observed that there were several binding elements of transcription factors involving inflammatory response in the region of HBXIP promoter, including STAT3, STAT5a and AP1. To further validate whether these transcription factors contributed to the activation of HBXIP promoter mediated by TNF-α, we constructed a series of mutant-type HBXIP promoters (Supplementary Figure 3A and Figure 3A). In addition, we constructed a random mutant type of HBXIP promoter, as a negative control. The sequence of the mutant is not associated with the STAT3, STAT5a and AP1. Our data revealed that the treatment with TNF-α could also activate HBXIP promoter with the random mutant, suggesting that the elements of STAT3, STAT5a and AP1 is significant in activation of HBXIP promoter (Supplementary Figure 3B). Further, we observed that TNF-α-induced activation of HBXIP promoter could be remarkably abolished when the binding site of STAT3 was mutated (Supplementary Figure 3A). As expected, the activities of HBXIP promoter with the mutation of STAT3 element could not be increased when the cells were treated with TNF-α (Figure 3B and Supplementary Figure 3C), indicating that STAT3 may activate HBXIP promoter mediated by TNF-α. In addition, knockdown of STAT3 by siRNA was capable of abolishing the event in the cells (Figure 3C and Supplementary Figure 3D). Chromatin immunoprecipitation (ChIP) assays revealed that STAT3 could bind to the promoter of HBXIP in MDA-MB-468 cells, which could be enhanced by the treatment with TNF-α (Figure 3D). The interference efficiency of STAT3 was confirmed by Western blot analysis in the cells (Figure 3E). Collectively, we conclude that TNF-α is able to increase the activities of HBXIP promoter through activating transcription factor STAT3.

### TNF-α enhances STAT3 phosphorylation *via* NF-κB and/or p38 signaling in activation of HBXIP promoter

**Figure 4:**
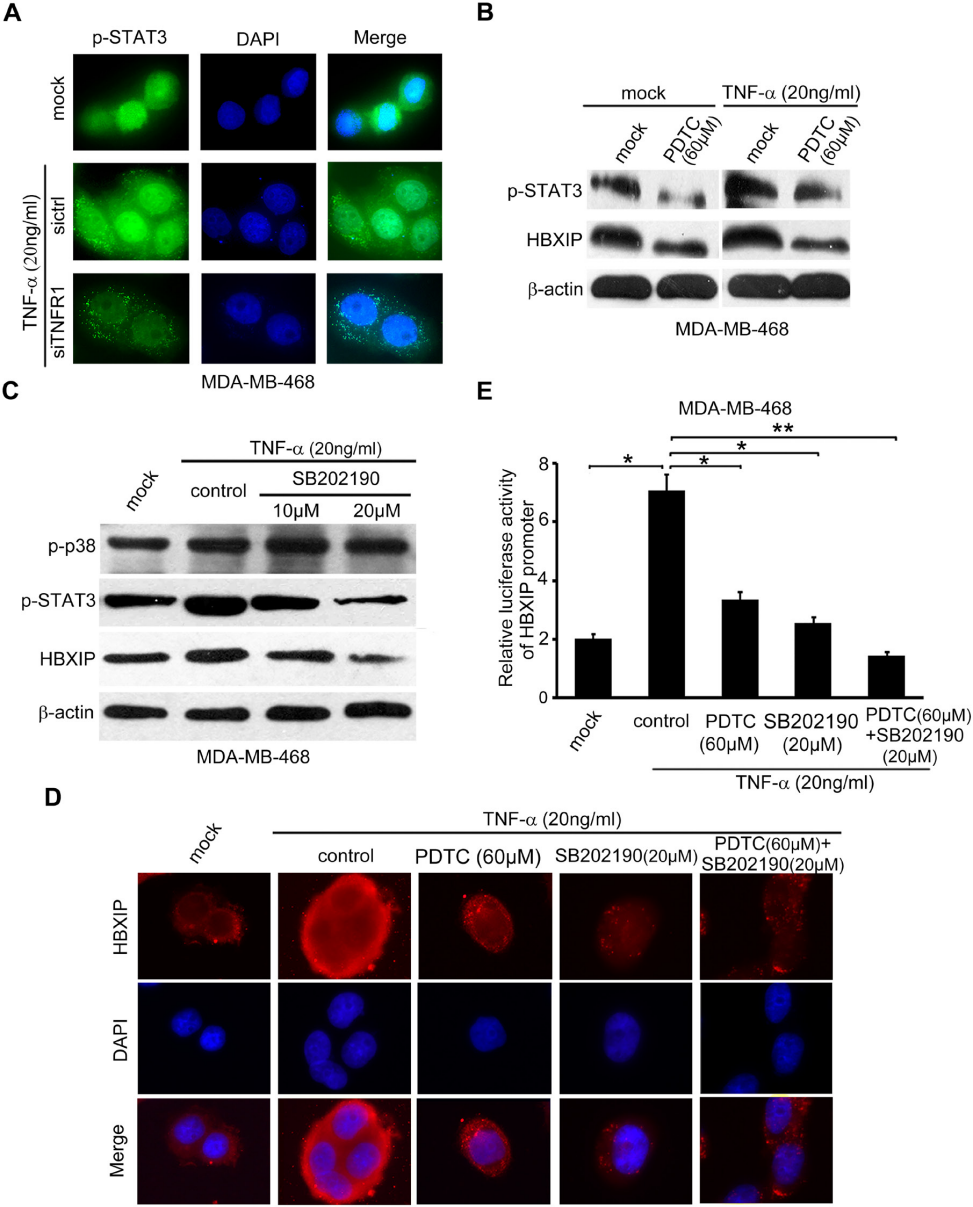
TNF-α enhances STAT3 phosphorylation via NF-κB and/or p38 signaling in activation of HBXIP promoter **(A)** Immunofluorescence and nucleocytoplasmic of p-STAT3 expression analysis were carried out in MDA-MB-468 cells treated with 20 ng/ml TNF-α and transiently transfected with siTNFR1 (200nM). Immunofluorescence images of p-STAT3 (green) were shown. DAPI staining (blue) was included to visualize the nucleus. **(B)** Western blot assays were applied for detecting the levels of p-STAT3 and HBXIP in MDA-MB-468 cells upon the treatment of 20 ng/ml TNF-α and/or 60 μM PDTC. **(C)** The levels of p-p38, p-STAT3, HBXIP were detected by Western blot analysis in MDA-MB-468 cells upon the treatment of 20 ng/ml TNF-α, 10 μM and 20 μM SB202190. **(D)** The immunofluorescence of HBXIP expression analysis was carried out in MDA-MB-468 cells treated with 20 ng/m TNF-α, 60 μM PDTC, 20 μM SB202190 or combimation of PDTC and SB202190. Immunofluorescence images of HBXIP (red) were shown. DAPI staining (blue) was included to visualize the nucleus. **(E)** The relative activities of HBXIP promoter were detected by dual luciferase reporter gene assays in MDA-MB-468 cells upon the treatment of 20 ng/m TNF-α coupled with 60 μM PDTC, 20 μM SB202190 or combination of PDTC and SB202190. Error bars represent ±s.d., *p < 0.05, **p < 0.01, Student's *t* test. All experiments were performed at least 3 times.

It has been reported that the phosphorylated STAT3 (p-STAT3) at site 705 tyrosine translocates into the nucleus and functions as a transcription factor to activate the expressions of target genes [36, 37]. Accordingly, immunofluorescence staining analysis showed that TNF-α was capable of promoting the translocation of p-STAT3 from cytoplasm to nucleus, which could be abolished by silencing TNFR1 (Figure 4A). NF-κB and p38/MAPK pathways are involved in the increase of STAT3 phosphorylation [17, 38]. Interestingly, we observed that the administration of PDTC, a specific inhibitor of NF-κB, could decrease the elevated-levels of p-STAT3 or HBXIP mediated by TNF-α in MDA-MB-468 and SK-BR cells (Figure 4B and Supplementary Figure 4A, 4B). In addition, TNF-α-increased nuclear retention of p-STAT3 could be attenuated by the treatment with PDTC in MDA-MB-468 cells (Supplementary Figure 4C, 4D). Moreover, the silencing of P65 significantly inhibited the TNF-α-induced HBXIP promoter activities in the cells (Supplementary Figure 4E), suggesting that NF-κB-activated STAT3 is involved in the up-regulation of HBXIP induced by TNF-α in breast cancer cells. Additionally, we demonstrated that TNF-α-increased p-STAT3 and HBXIP could be abolished by the treatment with SB202190, an inhibitor of p-p38 phosphokinase activity, in MDA-MB-468 and SK-BR3 cells (Figure 4C and Supplementary Figure 4F, 4G), suggesting that p-p38/STAT3 signaling is required for the TNF-α-induced HBXIP expression. Notably, we revealed that the combination treatment of PDTC and SB202190 led to the down-regulation of HBXIP relative to single use of PDTC or SB202190 in the cells (Figure 4D, 4E). Collectively, we conclude that TNF-α increases STAT3 phosphorylation *via* NF-κB and/or p38 signaling in activation of HBXIP promoter in breast cancer.

### TNF-α-elevated HBXIP up-regulates the expression of TNFR1 in breast cancer cells

**Figure 5:**
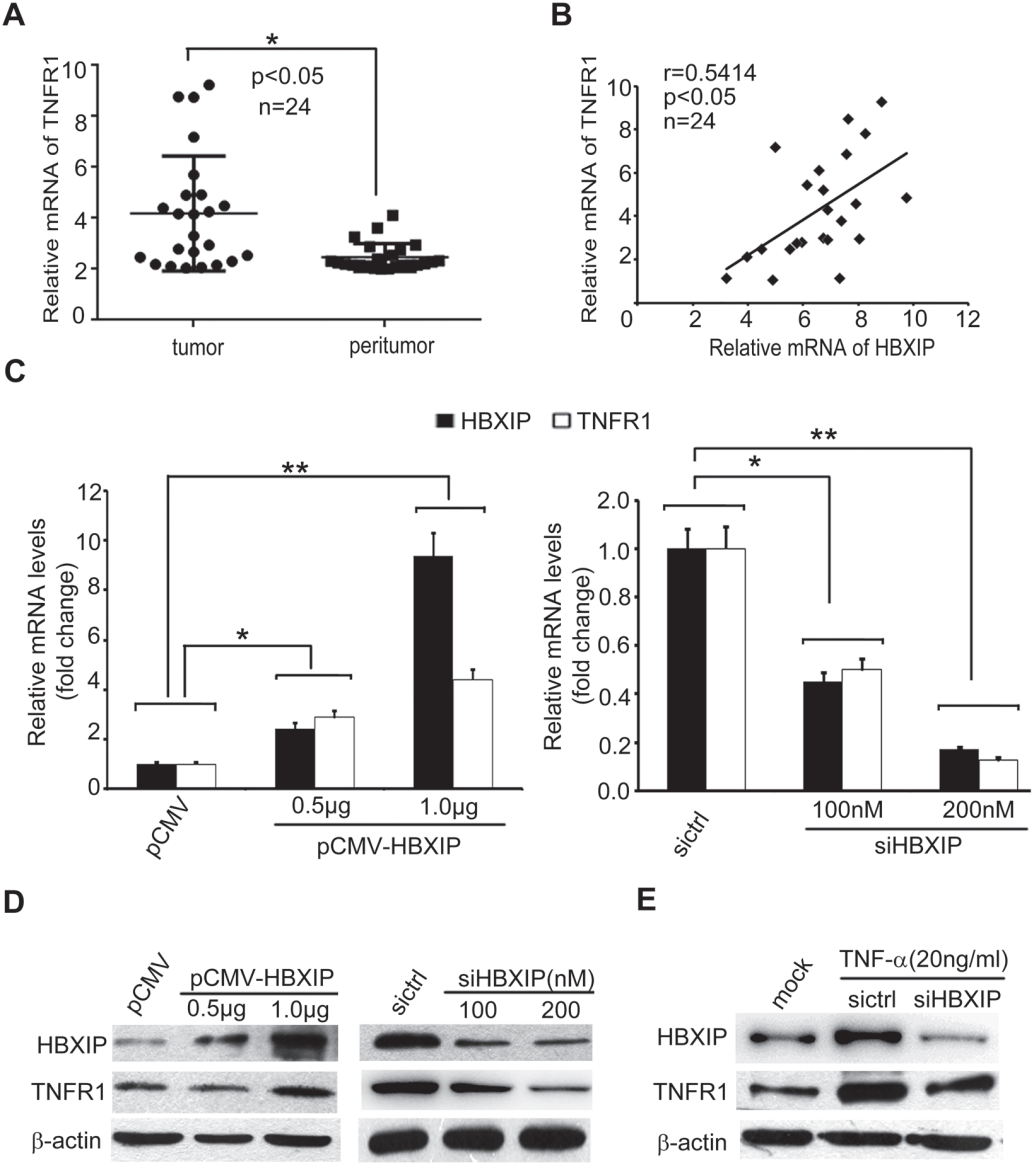
TNF-α-elevated HBXIP up-regulates the expression of TNFR1 in breast cancer cells **(A)** The expression levels of TNFR1 were detected by qRT-PCR analysis in clinical breast cancer tissues (n=24) (*p<0.05, Wilcoxon's signed-rank test). **(B)** The relative mRNA levels of HBXIP and TNFR1 were examined by qRT-PCR analysis in above samples (Spearman's correlation = 0.5414, *p < 0.05). **(C)** The relative fold changes of TNFR1 and HBXIP mRNA levels were detected by qRT-PCR analysis in MDA-MB-468 cells transiently transfected with pCMV-HBXIP or siHBXIP. **(D, E)** The expression levels of TNFR1 and HBXIP were examined by Western blot analysis in MDA-MB-468 cells transiently transfected with pCMV-HBXIP or siHBXIP and/or treated with 20 ng/ml TNF-α. Error bars represent ±s.d., *p < 0.05, **p < 0.01, Student's *t* test. All experiments were repeated at least 3 times.

As we know, the feedback regulation is frequently occurred in cancers [7, 11]. Our group has reported that HBXIP can promote lipid metabolism processes and MDM2/P53 in a feedback loop manner in breast cancer [28, 34]. Therefore, we speculated that HBXIP might be involved in the regulation of TNFR1 in a feedback manner. We observed that the mRNA levels of TNFR1 were higher in 24 cases of clinical breast cancer tissues relative to their corresponding peritumor tissues (Figure 5A and Supplementary Table 1). QRT-PCR analysis revealed that the expression levels of HBXIP were positively associated with those of TNFR1 in above cancer samples (Figure 5B). As expected, we observed that HBXIP overexpression could up-regulate TNFR1, and silencing of HBXIP by siRNA resulted in the down-regulation of TNFR1, at the levels of mRNA and protein in MDA-MB-468 and SK-BR3 cells in a dose dependent manner (Figure 5C, 5D and Supplementary Figure 5A-5D). Upon the administration of TNF-α, knockdown of HBXIP could inhibit the expression of TNFR1 in the cells (Figure 5E and Supplementary Figure 5E), suggesting that TNF-α is capable of up-regulating TNFR1 through activating HBXIP. Thus, we conclude that TNF-α-elevated HBXIP is able to up-regulate TNFR1 in breast cancer cells.

### TNF-α promotes the growth of breast cancer through HBXIP *in vitro* and *in vivo*

**Figure 6:**
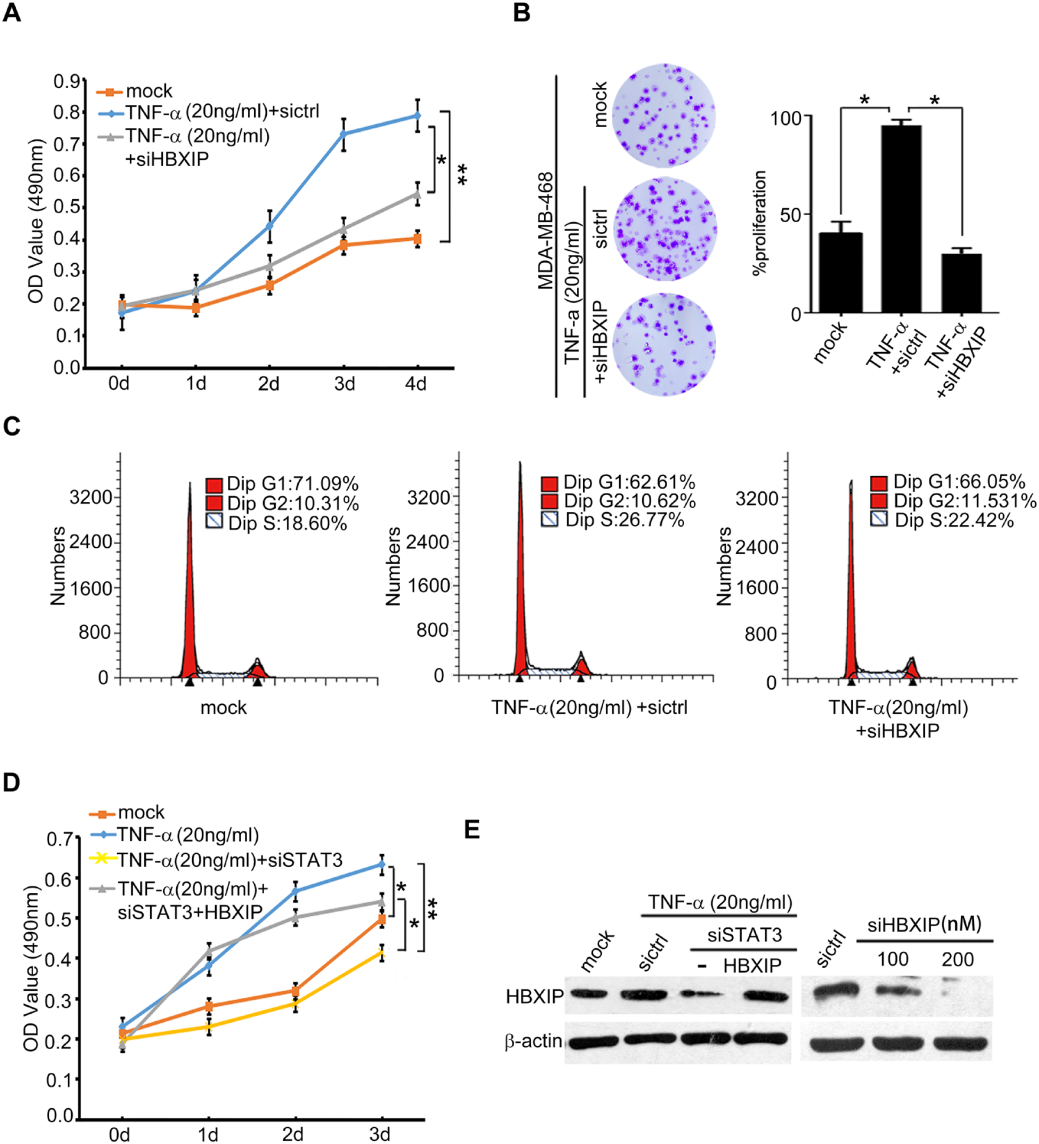
TNF-α promotes the growth of breast cancer through HBXIP *in vitro* **(A, B)** Effect of HBXIP knockdown on TNF-α-elevated cell proliferation was examined by MTT and colony formation assays in MDA-MB-468 cells transiently transfected with siHBXIP and treated with 20 ng/ml TNF-α. **(C)** Effect of HBXIP knockdown and TNF-α on cell cycle was measured by flow cytometry analysis in MDA-MB-468 cells. **(D)** Effect of STAT3 knockdown and HBXIP on TNF-α-enhanced cell proliferation was detected by MTT assays in MDA-MB-468 cells. **(E)** The expression levels HBXIP were validated by Western blot analysis in MDA-MB-468 cells. Error bars represent ±s.d., *p< 0.05, **p< 0.01, Student's *t* test. All experiments were repeated at least 3 times.

**Figure 7:**
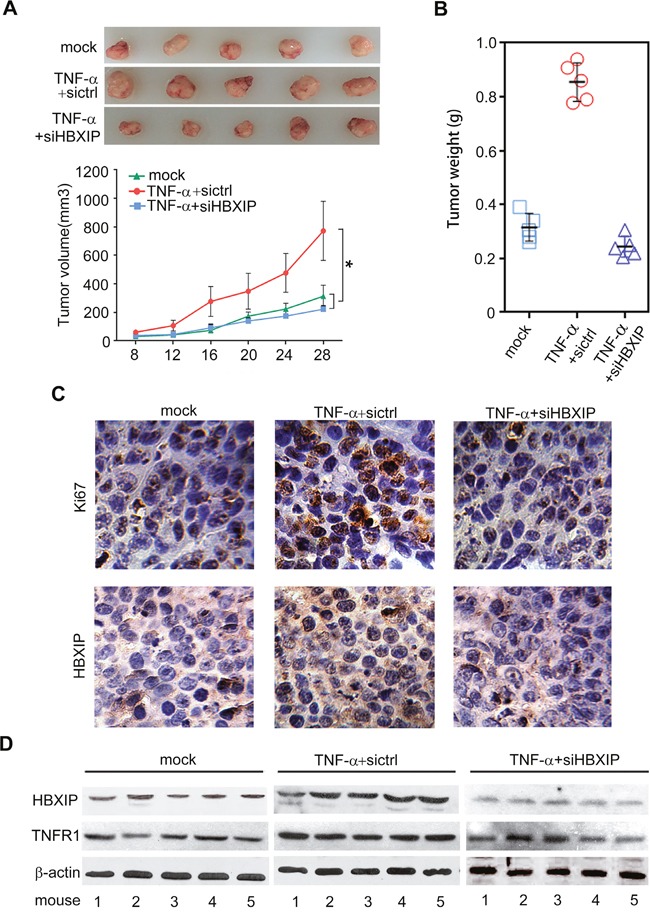
TNF-α promotes the growth of breast cancer through HBXIP *in vivo* **(A)** The tumors in each group were shown in up panel. The growth curve of tumors in nude mice transplanted with MDA-MB 468 cells was shown in down panel (n=5). **(B)** The tumor weight in each group was shown. **(C)** The expression of Ki67 and HBXIP was detected by immunohistochemistry assays in tumor tissues from mice. **(D)** The expression levels of HBXIP and TNFR1 were examined by Western blot analysis in the tumor tissues from mice. Error bars represent ±s.d., *p< 0.05, Student's *t* test. All experiments were applied at least 3 times.

Next, we evaluated whether TNF-α could promote the growth of breast cancer through HBXIP. MTT and colony formation assays revealed that knockdown of HBXIP significantly blocked the TNF-α-enhanced proliferation in MDA-MB-468 cells (Figure 6A, 6B). Moreover, flow cytometry analysis showed that TNF-α increased the percentage of cells in S phase, whereas silencing of HBXIP by siRNA could abolish the event (Figure 6C). Strikingly, MTT analysis showed that knockdown of STAT3 could abrogate TNF-α-accelerated cell proliferation. Conversely, the overexpression of HBXIP could rescue the inhibition caused by STAT3 knockdown in the cells treated with TNF-α (Figure 6D and Supplementary Figure 6), suggesting that TNF-α promotes the proliferation of breast cancer cells through STAT3/HBXIP signaling. The efficiency of interference or overexpression of HBXIP was validated by Western blot analysis in the cells (Figure 6E). Then, tumor xenograft assays showed that the treatment with TNF-α significantly promoted the growth of MDA-MB-468 cells in nude mice, whereas silencing of HBXIP remarkably attenuated the tumor growth (Figure 7A, 7B). Moreover, immunohistochemistry staining displayed that the expression levels of HBXIP and Ki67, a cell proliferation marker, were markedly increased in the tumor tissues from mice, but knockdown of HBXIP resulted in the opposite results (Figure 7C). In addition, Western blot analysis validated the expression levels of HBXIP and TNFR1 in the tumor tissues from mice (Figure 7D). Thus, we conclude that TNF-α can promote the growth of breast cancer through HBXIP *in vitro* and *in vivo*.

## DISCUSSION

The links between inflammation and cancer become a hot topic for cancer [2]. However, the mechanism underlying remains largely unknown. Cancer-related inflammatory factors contribute to the event [1, 5, 39]. Among those inflammatory factors, TNF-α/TNFR1-mediated signaling has been widely investigated [35]. Our group has reported that HBXIP as a key oncoprotein promotes the development of breast cancer [27, 28, 31, 33, 34]. In this study, we are interested in the effect of TNF-α on HBXIP in breast cancer.

Given that TNF-α could promote the development of head/neck squamaous cancer and gastric tumor [10, 11], we concerned whether TNF-α was involved in the progression of breast cancer. Interestingly, we found that the treatment with TNF-α could accelerate the proliferation of breast cancer cells. Moreover, our data ruled out the possibility of apoptosis induced by using 20 ng/ml TNF-α in the cells. Then, we found that the expression levels of TNF-α were positively associated with those of HBXIP in clinical breast cancer tissues. Interestingly, we demonstrated that TNF-α/TNFR1 could up-regulate HBXIP in breast cancer cells. TNF-α failed to up-regulate HBXIP cells when TNFR1 was knockdown by siRNA in the cells. It suggests that TNF-α is able to enhance the expression of the oncoprotein HBXIP through TNFR1 in breast cancer cells.

Next, we try to explore the mechanism by which TNF-α up-regulates HBXIP in breast cancer. It has been reported that STAT3 acts as an important transcription factor in cancers [36, 40]. The phosphorylated STAT3 functions in activation of its target genes transcription in the nucleus [36, 37, 41]. In this study, we validated that p-STAT3 contributed to the activation of HBXIP promoter mediated by TNF-α. As known, inflammatory cytokine TNF-α can initiate a complex of signaling cascades, such as NF-κB, JNK/AP1 and p38/MAPK signaling [7, 11, 42]. P-p38 can act as the phosphokinase to activate the phosphorylation of STAT3 [43]. To better understand the mechanism, we validated the effect of TNF-α on STAT3 phosphorylation. As expected, we observed that TNF-α could activate STAT3 *via* NF-κB and/or p38 signaling, leading to the up-regulation of HBXIP in breast cancer cells. TNF-α can also induce the expression of many oncoproteins through NF-κB or p38 signaling, such as JAG1, Fascin, VEGF and MMP2/MMP9, to promote the development of cancers [11, 35, 38, 44]. Therefore, we supposed that TNF-α might widely influence the expression of oncogenes including HBXIP in cancers. Moreover, we demonstrated that HBXIP could up-regulate the expression of TNFR1, forming a positive feedback loop of TNFR1/NF-κB (and/or p38)/p-STAT3/HBXIP/TNFR1. Functionally, we provided the evidence that the administration of TNF-α promoted the growth of breast cancer through HBXIP *in vitro* and *in vivo*. Thus, we conclude that TNF-α/TNFR1 activates the NF-κB (and/or p38)/STAT3/HBXIP signaling, leading to the development of cancer. Therapeutically, the oncoprotein HBXIP may serve as a target for breast cancer.

In summary, we present a model that inflammatory factor TNF-α contributes to the carcinogenesis through activating the oncoprotein HBXIP in breast cancer. In this model, TNF-α as a tumor promoter stimulates TNFR1, followed by NF-κB and/or p38/MAPK signaling leads to the activation of transcriptional factor STAT3, which results in the up-regulation of oncoprotein HBXIP. Strikingly, HBXIP up-regulates TNFR1 expression, forming a positive feedback loop of TNFR1/NF-κB (and/or p38)/STAT3/HBXIP/TNFR1 in breast cancer. Our finding provides new insights into the mechanism by which TNF-α stimulates HBXIP in the link between inflammation and cancer in breast cancer.

## MATERIALS AND METHODS

### Patient samples

Twenty-four cases of clinical breast tumor tissues and their corresponding peritumor tissues were obtained from Tianjin First Center Hospital (Tianjin, China) after surgical resection. Written consent approving the use of samples for research purposes was obtained from patients. The information of patients with breast cancer is presented in Supplementary Table 1. The study protocol was approved by the Institute Research Ethics Committee at Nankai University.

### Reagents, cell lines and cell culture

The reagents used in this study were SB202190 (Sigma, St. Louis, USA), PDTC (Sigma, St. Louis, USA), TNF-α (Peprotech, USA). Breast cancer cell lines MDA-MB-468 and SK-BR3 were cultured in DMEM medium (Gibco, Grand Island, NY), 10% fetal calf serum (FCS), 100 U/ml penicillin, and 100 μg/ml streptomycin in humidified 5% CO_2_ at 37°C.

### Chromatin immunoprecipitation (ChIP)

ChIP assays were performed in MDA-MB-468 cells according to the manufacturer's protocol (Epigentek Group Inc, Brooklyn, NY) as reported previously [45]. The promoter region of HBXIP including the STAT3 binding sites was amplified from the immunoprecipitated DNA samples with the specific primers. All primers are listed in Supplementary Table 2. All experiments were applied at least 3 times.

### Immunohistochemistry staining (IHC)

Immunohistochemistry staining of samples were performed as previously reported [46]. Anti-Ki67 (Cell Signaling Technology company, CST, USA)and anti-HBXIP (Santa Cruz Biotechnology, Santa Cruz, CA) were used [33, 47]. Categorization of immunostaining intensity was performed by 3 independent observers.

### Tumor xenograft in mice

Nude mice were nursed and treated according to the guidelines established by the National Institutes of Health Guide for the Care and Use of Laboratory Animals. The animal transplantation was conducted according to the Declaration of Helsinki. Tumor transplantation was performed in nude mice. In brief, mock group with MDA-MB-468 cells was added into the control solvent (BSA, bovine serum albumin) and the test group with MDA-MB-468 cells was treated with 20 ng/ml TNF-α coupled with silencing of HBXIP or si-control every two days for 5 times. Cells were harvested and re-suspended at 2× 10^7^ per ml with sterile phosphate-buffered saline. Three groups of 4-week-old female BALB/c athymic nude mice (Experiment Animal Center of Peking, China) (each group, n=5) were subcutaneously injected at the mouse shoulder with 0.1 ml of the cell suspensions. Tumor volume was measured 8 days after injection and then every 4 days. Tumor volume (V) = (L× W^2^) × 0.5 was calculated by measuring the length (L) and width (W) with calipers. After 30 days, mice were sacrificed and the tumors were excised and measured for further research.

### Dual luciferase reporter gene assays

Adherent cells were seeded into 24-well plates to make sure the cell confluence up to 60-80%, and then were cotransfected with the plasmids containing wild type or mutant of HBXIP promoter or pGL3-Basic as a negative control, and the pRL-TK plasmid (Promega, Madison, WI, USA) which was used as internal normalization. Cell extracts were harvested after 24 hours and lysed by using lysis buffer (Promega, Madison, WI, USA). Dual luciferase reporter gene assays were implemented using the Dual-Luciferase Reporter Assay System (Promega, Madison, WI, USA) as the manufacture's procedures. All experiments were repeated at least 3 times.

### Plasmid construction and small interference RNA (siRNA)

PGL3-Basic, pCMV-tag2B, pGL3-HBXIP, pRL-TK (Promega, Madison, WI, USA) and pCMV-HBXIP, pcDNA3.0, pcDNA3.0-HBXIP were kept in our lab. The mutants of STAT3, STAT5a, and AP1 in HBXIP promoter were cloned into the plasmid pGL3-basic. All primers and siRNA sequence were listed in Supplementary Table 2.

### RNA extraction, RT-PCR and quantitative real-time PCR (qRT-PCR)

Total RNA was extracted from the cells (or tissues) using Trizol (Invitrogen, Carlsbad, CA, USA) according to the manufacturer′s procedures. Reverse transcription was performed using poly (A)-tailed total RNA and reverse transcription primer with ImPro-II Reverse Transcriptase (Promega, Madison, WI, USA), according to the manufacturer′s protocols. RT-PCR was applied as previously reported [48]. The qRT-PCR was performed according to the instructions of Fast Start Universal SYBR Green Master (Rox) (Roche Diagnostics GmbH Mannheim, Germany). All primers were listed in Supplementary Table 2. GAPDH was used as the control.

### Western blot analysis

Western blot analysis was used as described previously [49]. Primary antibodies used in this study were rabbit anti-HBXIP antibody (Santa Cruz Biotechnology, Santa Cruz, CA, USA), rabbit anti-Ki67 antibody (Cell Signaling Technology company, CST, USA), mouse anti-**β**-actin antibody (Sigma, St. Louis, USA), rabbit anti-STAT3 antibody (Immunoway Biotechnology Company, USA), rabbit anti-Phospho-STAT3 antibody (Immunoway Biotechnology Company, USA) [50], rabbit anti-Phospho-p38 antibody (Cell Signaling Technology company, CST, USA) [51] and mouse anti-TNFR1 antibody (Proteintech Group, Chicago, IL, USA) [52], rabbit anti-P65 antibody (Proteintech Group, Chicago, IL, USA).

### Immunofluorescence staining

Cells were seeded on acid-treated glass cover slips, then cells were fixed 20 minutes with ice-cold 4% paraformaldehyde, washed three times with phosphate-buffered saline (PBS), 5 minutes per time on the table concentrator. Then, cells were permeabilized with 0.1% Triton X-100 in PBS for 20 minutes. After washing three times with PBS, samples were blocked in PBS containing 5% BSA for 1 hour, which then were incubated with primary antibodies for 2 hours at room temperature. After washed three times with PBS, the cells were incubated with fluorophore-conjugated secondary antibody (DAKO, Denmark) and executed for DAPI staining. Washing again, slides were mounted with 90% glycerol and observed under fluorescence microscope (Zeiss Axio Imager Z1, Germany). The cells were observed in three randomly selected fields.

### 3-(4,5-dimethylthiazol-2-yl)-2,5 diphenyltetrazolium bromide (MTT) assays

MDA-MB-468 cells were seeded onto 96-well plates (1000 cells/well) for 24 hours before transfection or other treatments. Then 3-(4,5-dimethylthiazol-2-yl)-2,5 diphenyltetrazolium bromide (MTT) assays were used to assess cell proliferation every day from the first day until the third day or fourth day after transfection.

### EdU assays

Five-ethynyl-2’-deoxyuridine (EdU) incorporation assays were carried out using the Cell-Light TM EdU imaging detecting kit according to the manufacturer's instructions (RiboBio, Guangzhou, China).

### Flow cytometry analysis

The flow cytometry analysis protocol was used as described previously [18]. MDA-MB-468 cells were plated in 6-well plates at 1× 10^5^ cells per dish and maintained in serum free DMEM for 48h and then cells were harvested and stained with propidium iodide or Annexin-V.

### Colony formation assays

For clonogenicity analysis, 48 hours after transfection, 1000 viable transfected cells were placed in 6-well plates and maintained in complete medium for 2 weeks. Colonies were fixed with methanol and stained with crystal violet.

### Statistical analysis

Each experiment was repeated at least 3 times. Statistical significance was assessed by comparing mean values (± sd) using a Student's *t* test for independent groups and was assumed for *p*<0.05 (*), *p*<0.01 (**), *p*<0.001 (***) and not significant (ns). The expression levels of TNF-α and TNFR1 in breast cancer tissues and their corresponding peritumor tissues were analyzed using the Wilcoxon signed-rank test. Pearson's correlation coefficient was used to determine the correlation between HBXIP and TNF-α or TNFR1 mRNA levels in breast cancer tissues.

## SUPPLEMENTARY MATERIALS FIGURES AND TABLES


