# Supplementary material for: Inflammatory factor TNF-α promotes the growth of breast cancer via the positive feedback loop of TNFR1/NF-κB (and/or p38)/p-STAT3/HBXIP/TNFR1
Source: Oncotarget. 2017 Apr 6;8(35):58338–52. doi: 10.18632/oncotarget.16873 (PMC5601656; doi:10.18632/oncotarget.16873)
Supplement: Supplementary file 1 [file oncotarget-08-58338-s001.pdf]

Inflammatory factor TNF-α promotes the growth of breast cancer via the positive feedback loop of TNFR1/NF-κB (and/or p38)/p-STAT3/HBXIP/TNFR1

SUPPLEMENTARY MATERIALS

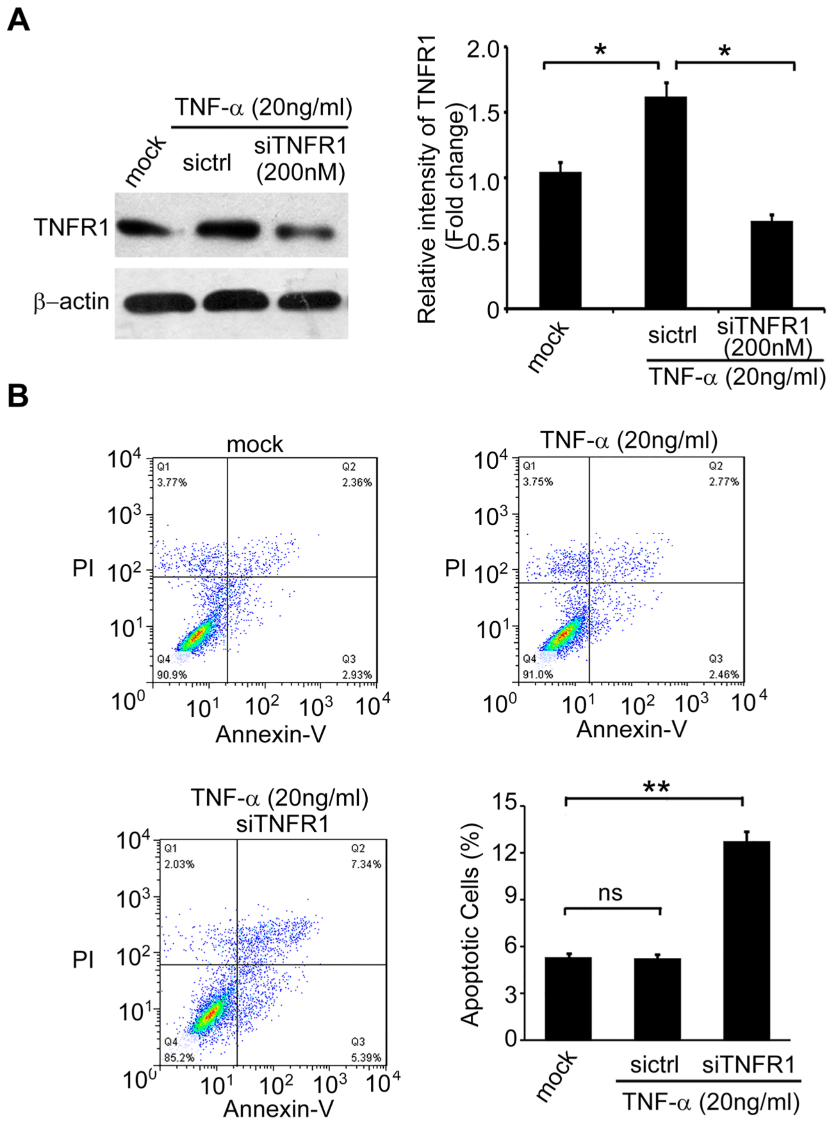

Supplementary Figure 1: TNF-α administration is able to promote the proliferation of breast cancer cells through activating TNFR1. (A) The expression level of TNFR1 was detected by Western blot analysis in MDA-MB-468 cells treated with TNF-α and 200 nM siTNFR1. **(B)** Annexin-V/PI staining of TNFR1 knockdown coupled with TNF-α treatment in SK-BR3 cells analyzed by FACS. Quantification of apoptotic cells, numbers represent the sum of early and late apoptotic cells. Error bars represent ±s.d., *$p < 0.05$, **$p < 0.01$, Student's *t* test. All experiments were repeated at least 3 times.

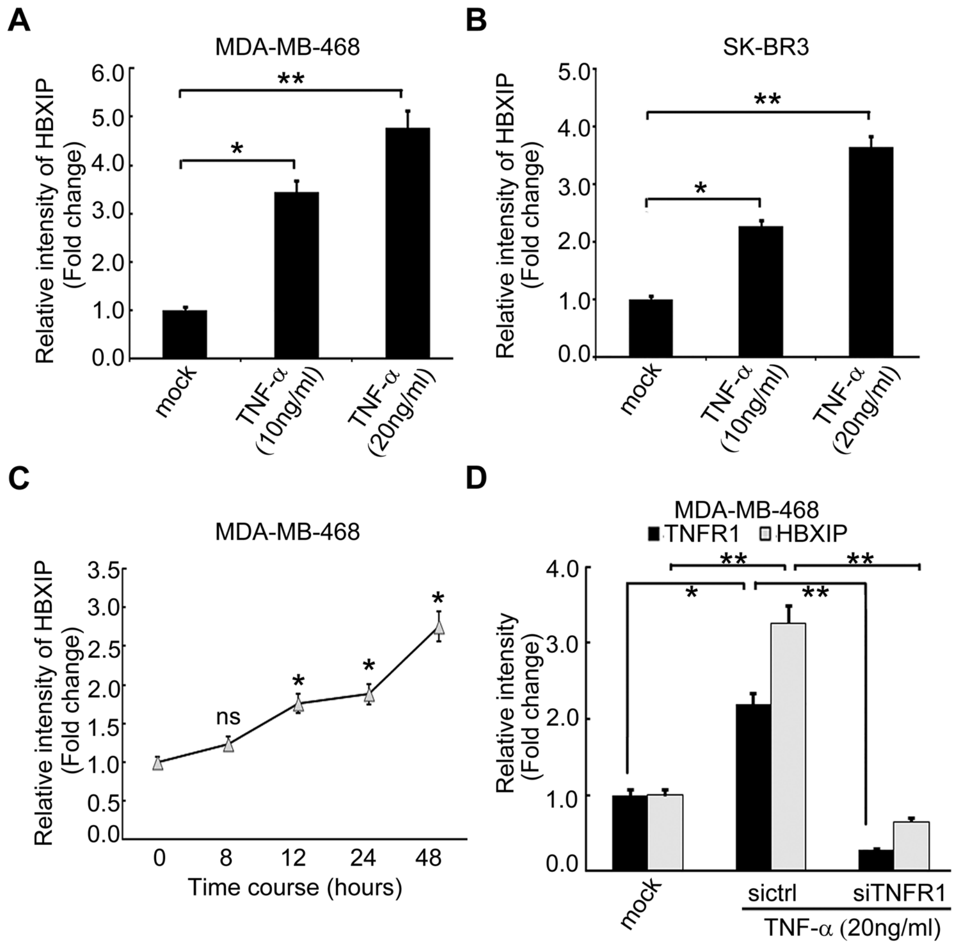

Supplementary Figure 2: TNF-α is positively correlated with HBXIP in clinical breast cancer tissues and up-regulates HBXIP in breast cancer cells. (A, B) Quantification of relative intensity of HBXIP protein levels analyzed by Western blot assays in MDA-MB-468 and SK-BR3 cells treated with 10 ng/ml, 20 ng/ml TNF-α, respectively. **(C)** Quantification of relative intensity of HBXIP protein levels analyzed by Western blot assays in MDA-MB-468 cells time dependently treated with 20 ng/ml TNF-α. **(D)** Quantification of relative intensity of HBXIP and TNFR1 protein levels analyzed by Western blot assays in MDA-MB-468 cells treated with 20 ng/ml TNF-α and transiently transfected with TNFR1 siRNA. Error bars represent ±s.d., *$p < 0.05$, **$p < 0.01$, Student's t test. All experiments were repeated at least 3 times.

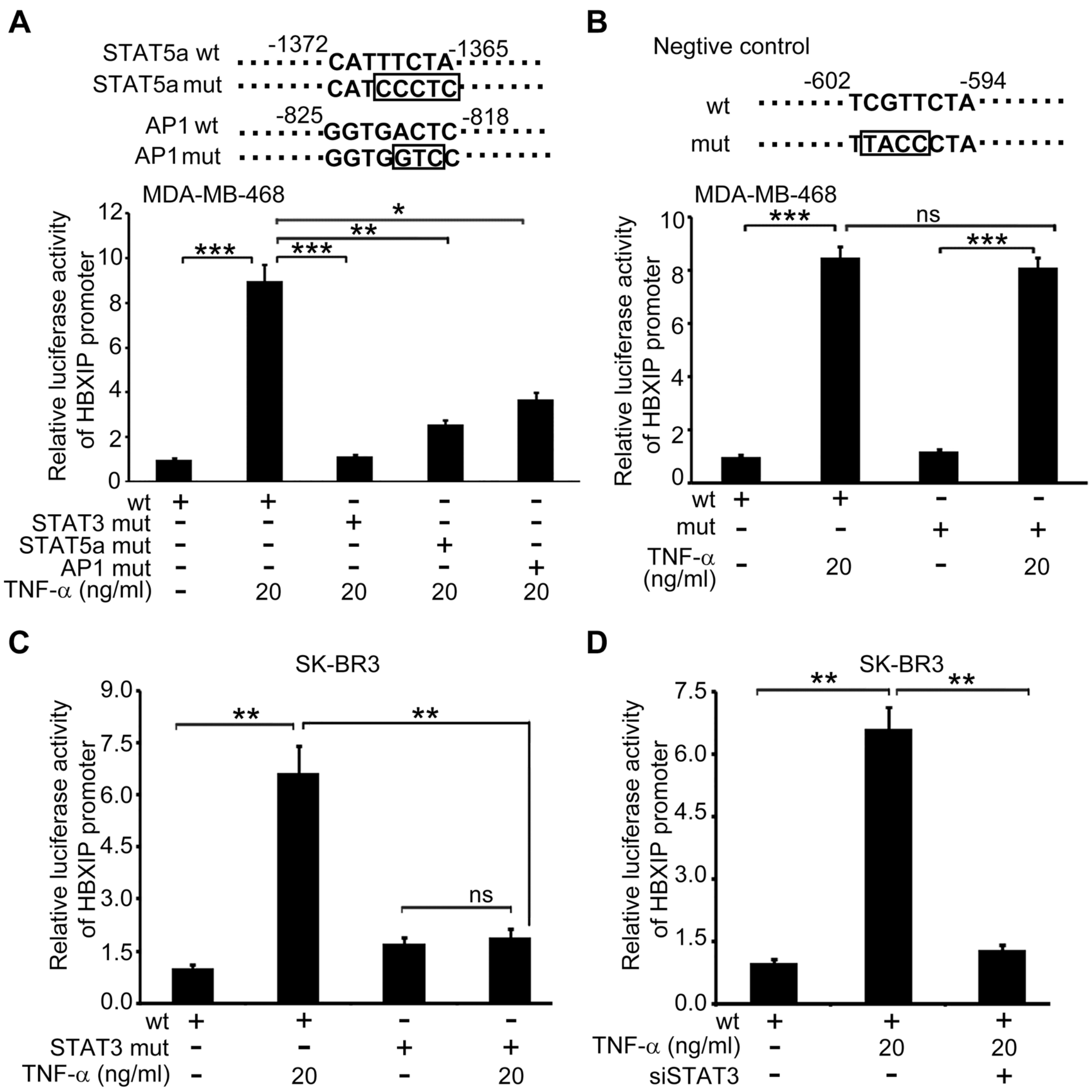

Supplementary Figure 3: TNF-α activates HBXIP promoter through transcription factor STAT3. (A) The up panel showed the mutation of binding sites of STAT5a and AP1 in the promoter of HBXIP. The down panel showed that the relative activities of HBXIP promoter were measured by dual luciferase reporter gene assays in MDA-MB-468 cells. **(B)** The up panel showed the mutation of binding sites of negative control in the promoter of HBXIP. The down panel showed that the relative activities of HBXIP promoter were measured by dual luciferase reporter gene assays in MDA-MB-468 cells. **(C)** The relative activities of HBXIP promoter of containing wild type (wt) or mutant type of STAT3 (STAT3 mut) binding site were detected by luciferase reporter gene assays in SK-BR3 cells administrated with 20 ng/ml TNF-α. **(D)** The relative activities of the promoter of HBXIP were detected by luciferase reporter gene assays in SK-BR3 cells administrated with 20 ng/ml TNF-α and transiently transfected with STAT3 siRNA. Error bars represent ±s.d., $*p < 0.05$, $**p < 0.01$, $***p < 0.001$, Student's *t* test. All experiments were repeated at least 3 times.

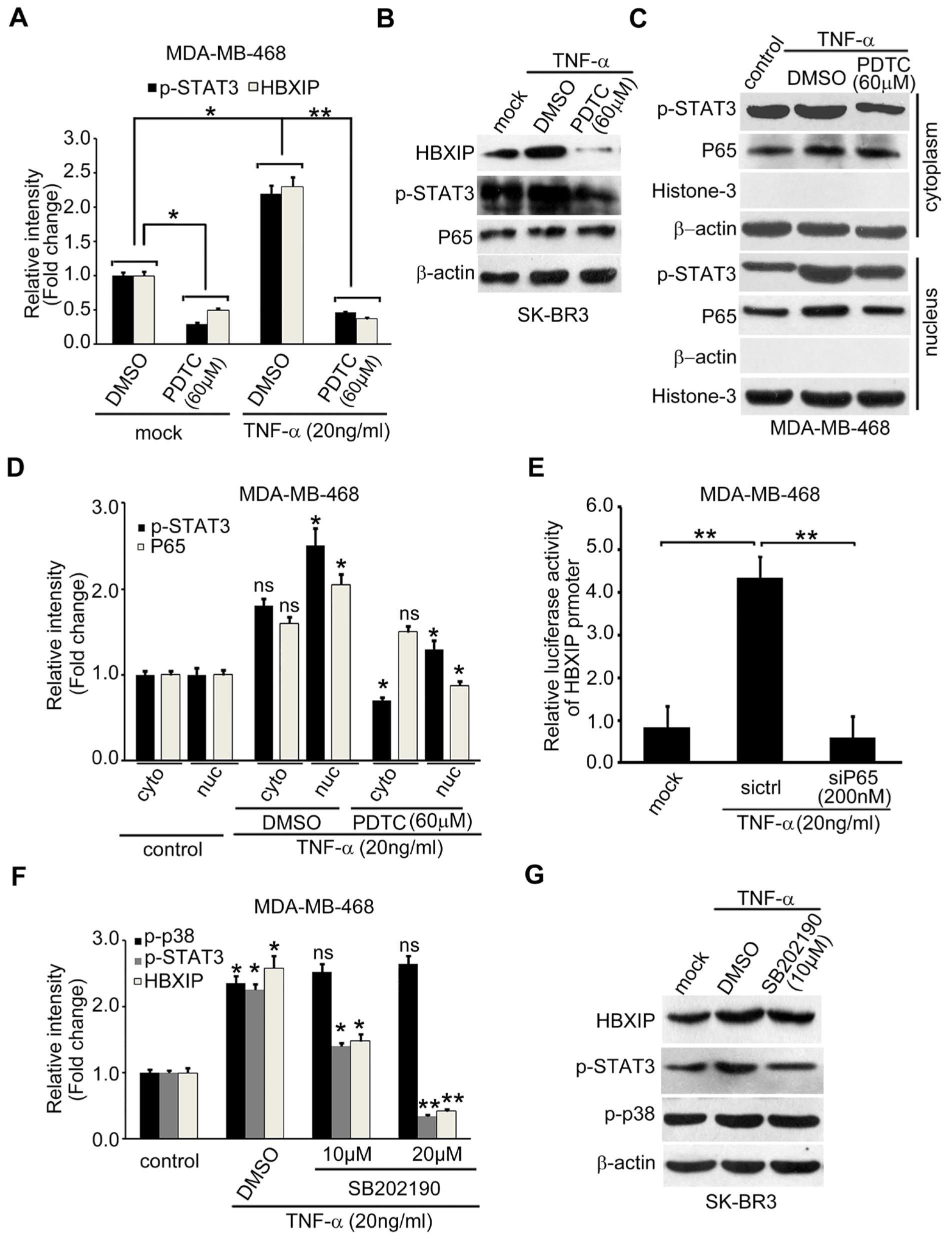

Supplementary Figure 4: TNF-α enhances STAT3 phosphorylation via NF-κB and/or p38 signaling in activation of HBXIP promoter. **(A)** Quantification of relative intensity of HBXIP and p-STAT3 protein levels analyzed by Western blot assays in MDA-MB-468 cells treated with 20 ng/ml TNF-α coupled with DMSO or PDTC treatment. **(B)** Western blot assays were applied for detecting the levels of p-STAT3 and HBXIP in SK-BR3 cells upon the treatment of 20 ng/ml TNF-α and 60 μM PDTC. **(C)** The nucleocytoplasmic analysis of p-STAT3 and P65 expression was determined by Western blot analysis in MDA-MB-468 cells added into 20 ng/ml TNF-α coupled with DMSO or 60 μM PDTC. **(D)** Quantification of relative intensity of P65 and p-STAT3 protein levels in MDA-MB-468 cells treated with 20 ng/ml TNF-α coupled with PDTC treatment ("cyto" means cytoplasm, "nuc" means nucleus) (cyto-DMSO vs cyto-control, cyto-PDTC vs cyto-DMSO; nuc-DMSO vs nuc-control, nuc-PDTC vs nuc-DMSO). **(E)** The relative activities of HBXIP promoter were measured by dual luciferase reporter gene assays in MDA-MB-468 cells treated with 20 ng/ml TNF-α coupled with silence of P65. **(F)** Quantification of relative intensity of HBXIP and p-STAT3 protein levels analyzed by Western blot assays in MDA-MB-468 cells treated with 20 ng/ml TNF-α coupled with DMSO or SB202190 administration (DMSO vs control, 10 μM-SB202190 vs DMSO, 20 μM-SB202190 vs DMSO). **(G)** The protein levels of HBXIP and p-STAT3 were analyzed by Western blot assays in SK-BR3 cells treated with 20 ng/ml TNF-α coupled with DMSO or SB202190 administration. Error bars represent ±s.d., *$p < 0.05$, **$p < 0.01$, Student's t test. All experiments were performed at least 3 times.

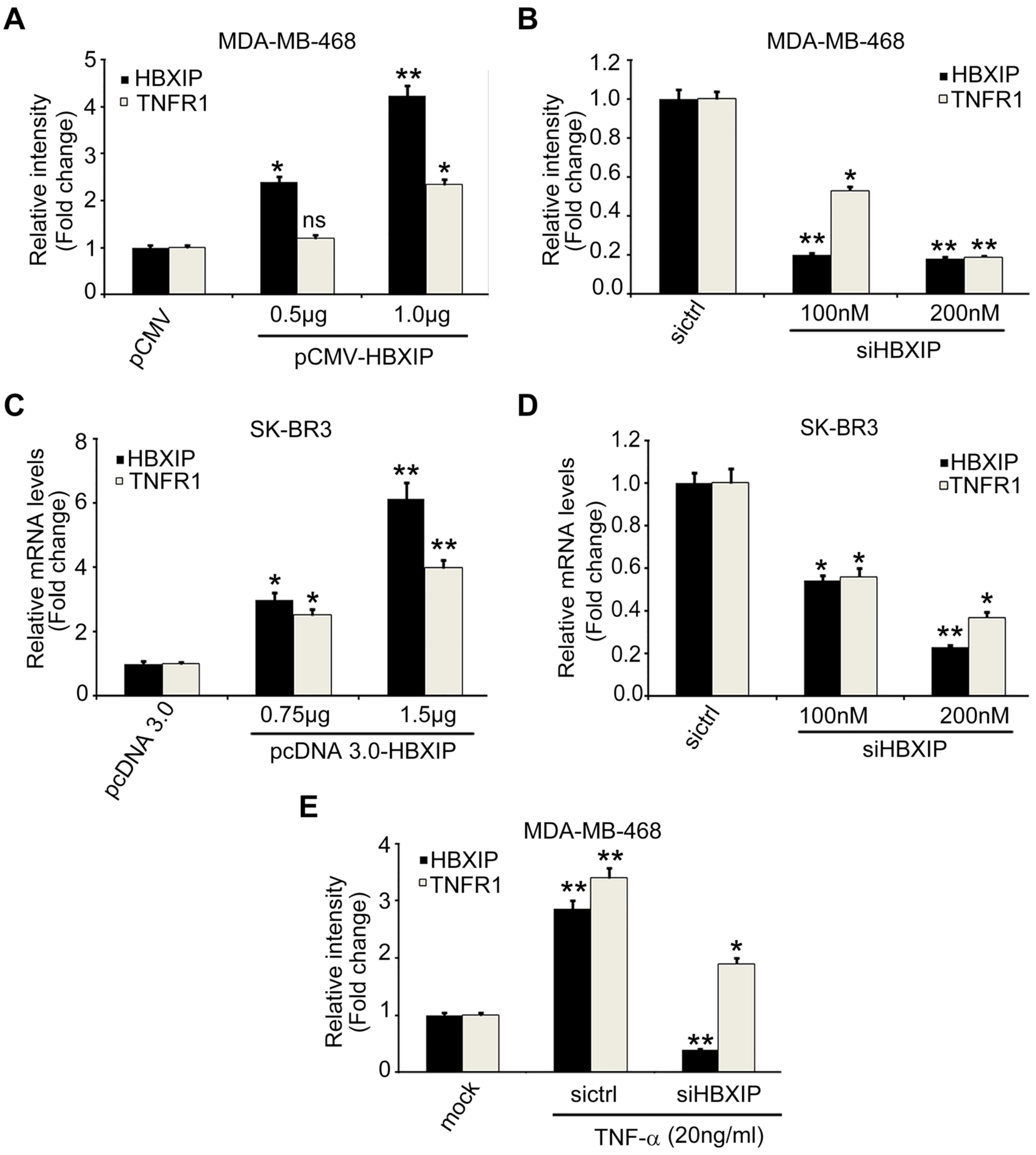

Supplementary Figure 5: TNF-α-elevated HBXIP up-regulates the expression of TNFR1 in breast cancer cells. (A, B) Quantification of relative intensity of HBXIP and TNFR1 protein levels analyzed by Western blot assays in MDA-MB-468 cells transiently transfected with pCMV-HBXIP or siHBXIP. **(C, D)** The relative fold changes of TNFR1 and HBXIP mRNA levels were detected by qRT-PCR analysis in SK-BR3 cells transiently transfected with pcDNA3.0-HBXIP or siHBXIP. **(E)** Quantification of relative intensity of HBXIP and TNFR1 protein levels analyzed by Western blot assays in MDA-MB-468 cells transiently transfected with siHBXIP and treated with 20 ng/ml TNF-α. Error bars represent ±s.d., *$p < 0.05$, **$p < 0.01$, Student's *t* test. All experiments were performed at least 3 times.

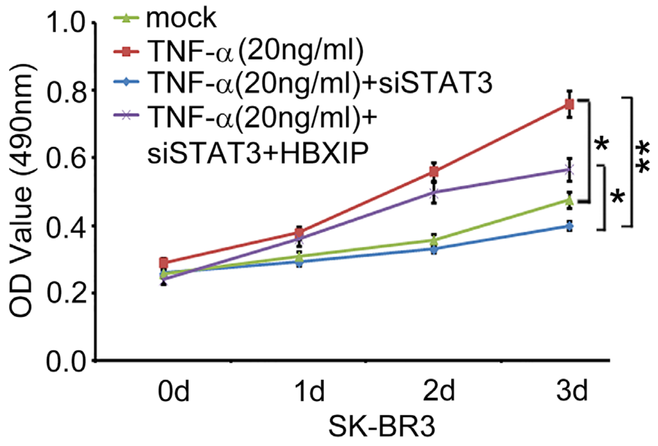

Supplementary Figure 6: TNF-α promotes the growth of breast cancer through HBXIP *in vitro*. Effect of STAT3 knockdown and HBXIP over-expression on TNF-α-enhanced cell proliferation was detected by MTT assays in SK-BR3 cells. Error bars represent ±s.d., $*p < 0.05$, $**p < 0.01$, Student's t test. All experiments were performed at least 3 times.

Supplementary Table 1: Clinical characteristics of breast tumor and peritumor samples

No.	Age	Sex	Organ	Pathology diagnosis	Grade
01	43	F	Breast	Nonspecific infiltrating ductal carcinoma	I
02	40	F	Breast	A little nonspecific infiltrating ductal carcinoma	I
03	54	F	Breast	Nonspecific infiltrating ductal carcinoma	I
04	64	F	Breast	Nonspecific infiltrating ductal carcinoma	I
05	41	F	Breast	Little nonspecific infiltrating ductal carcinoma	I
06	52	F	Breast	Little nonspecific infiltrating ductal carcinoma	I
07	44	F	Breast	Nonspecific infiltrating ductal carcinoma	II
08	60	F	Breast	Little nonspecific infiltrating ductal carcinoma	II
09	56	F	Breast	Little nonspecific infiltrating ductal carcinoma	I-II
10	55	F	Breast	Nonspecific infiltrating ductal carcinoma	II
11	51	F	Breast	Nonspecific infiltrating ductal carcinoma	II
12	74	F	Breast	Nonspecific infiltrating ductal carcinoma	II
13	56	F	Breast	Nonspecific infiltrating ductal carcinoma	II
14	42	F	Breast	Nonspecific infiltrating ductal carcinoma	II
15	57	F	Breast	Nonspecific infiltrating ductal carcinoma	II
16	50	F	Breast	Nonspecific infiltrating ductal carcinoma	II
17	45	F	Breast	Nonspecific infiltrating ductal carcinoma	II
18	35	F	Breast	Nonspecific infiltrating ductal carcinoma	II
19	38	F	Breast	Nonspecific infiltrating ductal carcinoma	II
20	58	F	Breast	Little nonspecific infiltrating ductal carcinoma	III
21	40	F	Breast	Nonspecific infiltrating ductal carcinoma	II
22	70	F	Breast	Nonspecific infiltrating ductal carcinoma	II
23	23	F	Breast	Nonspecific infiltrating ductal carcinoma	II
24	44	F	Breast	Nonspecific infiltrating ductal carcinoma	I-II

Note: “-” No grading available.

Supplementary Table 2: List of primers used in this paper

Genes	Primers	Sequence (5′-3′)
**Primers for HBXIP promoter**		
HBXIP	Forward	GGTACCGGGTGAGGCTTACCAATT
	Reverse	CCAAGCTTGAGGCGCGCACTACTCACGT
**Primers for HBXIP promoter mutant type**		
STAT3	Forward	GTGTGCGTCTTCTTCCCTAC
	Reverse	GCTTGTAGGGAAGAAGACGC
STAT5a	Forward	TCCATCCTCAGTAACTTATAAGGA
	Reverse	AGTTACTGAGGATGGAATGACT
AP1	Forward	CATGCTAGGCGTGGTGGT
	Reverse	GCTGGACCACCACGCCTA
Negative control	Forward	ATTACCCTACTGCACTACAGCCT
	Reverse	TAATGGGATGACGTGATGTCGGA
**Primers for RT-PCR**		
	Reverse	CTGCATGAGCAGGACCTCCAG
HBXIP	Forward	CTTGGAGCAGCACTTGGAAGA
	Reverse	ATGCCATCGTGTTTCTGGATC
GAPDH	Forward	AACGGATTTGGTCGTATTG
	Reverse	CCTGGTATGAGCCCATCTATC
TNF-α	Forward	CTGCCCGGGAAATGCTGCGA
	Reverse	CGAAGTGGTGGTCTTGTTGC
TNFR1	Forward	CCTGGTCATTTTCTTTGGTC
	Reverse	GAGGGCTGTCGCAAGGAT
**Primers for ChIP**		
HBXIP	Forward	CCCTCACCATAATAAGCACC
	Reverse	CCCTCACCATAATAAGCACC
**siRNA duplexes**		
HBXIP siRNA	Sense	CGGAAGCGCAGUGAUGUUUdTdT
TNFR1 siRNA	Sense	GGAGCTTACTTGTATGATGAT
STAT3 siRNA	Sense	CATCTGCCTAGATCGGCTA
P65 siRNA	Sense	GCCCUAUCCCUUUACGUCA
Control siRNA	Sense	UUCUCCGAACGUGUCACGUdTdT
